# Immunomodulatory actions of vitamin D in various immune-related disorders: a comprehensive review

**DOI:** 10.3389/fimmu.2023.950465

**Published:** 2023-07-14

**Authors:** Amirhossein Ghaseminejad-Raeini, Ali Ghaderi, Amirmohammad Sharafi, Behrad Nematollahi-Sani, Maryam Moossavi, Afshin Derakhshani, Gholamreza Anani Sarab

**Affiliations:** ^1^ Students Scientific Research Center, Tehran University of Medical Sciences, Tehran, Iran; ^2^ Nanobiology and Nanomedicine Research Center, Shiraz University of Medical Sciences, Shiraz, Iran; ^3^ Laboratory of Experimental Pharmacology, IRCCS Istituto Tumori Giovanni Paolo II, Bari, Italy; ^4^ Cellular and Molecular Research Committee, Birjand University of Medical Sciences, Birjand, Iran

**Keywords:** Vitamin D, autoimmunity, innate immunity, acquired immunity, autoimmune disorders

## Abstract

For many years, vitamin D has been acknowledged for its role in maintaining calcium and phosphate balance. However, in recent years, research has assessed its immunomodulatory role and come up with conflicting conclusions. Because the vitamin D receptor is expressed in a variety of immune cell types, study into the precise role of this molecule in diseases, notably autoimmune disorders, has been made possible. The physiologically activated version of vitamin D also promotes a tolerogenic immunological condition in addition to modulating innate and acquired immune cell responses. According to a number of recent studies, this important micronutrient plays a complex role in numerous biochemical pathways in the immune system and disorders that are associated with them. Research in this field is still relatively new, and some studies claim that patients with severe autoimmune illnesses frequently have vitamin D deficiencies or insufficiencies. This review seeks to clarify the most recent research on vitamin D’s immune system-related roles, including the pathophysiology of major disorders.

## Introduction

Vitamin D is an attractive molecule that has received particular attention recently. It is a major calcium homeostasis and bone metabolism modulator, increasing phosphorus and calcium absorption from the intestine, decreasing their excretion from the kidney, and promoting osteogenesis (1). It is also one of the crucial immune system regulator hormones that affect immune responses. Vitamin D acts as a pro-survival molecule. This vitamin defends cells against damaging signals by inhibiting inflammatory responses, such as changing the pathways through which T-helper-2 (Th2), M2 macrophages, and regulatory T cells (Treg) differentiate in order to maintain energy and redox homeodynamics by supplying a tolerogenic state (2). Vitamin D insufficiency continues to be one of the most common causes of osteoporosis, muscle weakness, falling fractures (particularly in the elderly), numerous malignancies, metabolic syndrome, cardiovascular illnesses, immune-mediated diseases (including autoimmune diseases), and infections (3–8). Ultraviolet (UV) rays that reach the epidermal layer of the skin and regular diet are the two main sources of vitamin D. That suggests that it should not be viewed solely as a vitamin: pro-hormone is the proper category (9). The normal range for vitamin D is still disputed among different nations. However, it is generally acknowledged that an adult can deal with a lower limit of 50–75 nmol/L. Numerous problems, including impaired bone metabolism, falling risk, various myopathies, and immune system dysregulation, are linked to low vitamin D levels (9–12).

The amount of vitamin D the body receives from food sources is typically insufficient, and these abundant sources are also scarce. These are the main factors that contribute to vitamin D deficiency (13). Vitamin D is present in small amounts in butter, peanuts, and eggs, but is present in large quantities in some foods like fish liver oil. Additionally, both cow milk and breast milk are deficient in vitamin D (1, 14). Despite the fact that vitamin D can be gained through diet, the skin is still the primary source of this pro-hormone. Seven-dehydrogenated cholesterol undergoes spontaneous transformation into vitamin D3 under the influence of UV radiation (13). The primary molecule that carries vitamin D and its subsequent metabolites in the blood is vitamin D binding protein (DBP). This vitamin needs to be hydroxylated twice in order to be physiologically active (14). The expression of the enzyme 1-hydroxylase, which converts 25-hydroxyvitamin D3 (25-OH D3) (precursor) to 1,25-(OH)2 D3 enabling immune microenvironments to respond to this substance in antigen-presenting cells, is one of the factors contributing to the recent interest in the immune-related function of vitamin D (13). It is initially hydroxylated by hydroxylase and several cytochrome P450 isoforms on the 25th carbon. Vitamin D status is mostly tracked by measuring 25-OH D3, which has a 2-week half-life and is the primary form of the vitamin in circulation.

As a result of the CYP27B1 gene expression, 1-alpha-hydroxylase carries out the second stage of hydroxylation in the kidney, skin, and immune cells, resulting in the production of 1,25-(OH)2 D3 or calcitriol (15, 16). The binding of vitamin D to its nuclear receptors, known as vitamin D receptors or VDRs, inside target cells controls gene expression. VDR is also found in non-classical tissues like the brain, eye, heart, pancreatic islet beta cells, and immune cells. The principal mechanism of action of vitamin D in those tissues is metabolism (17). The identification of VDR in immune cells is related to the putative function of vitamin D in controlling immunological responses, cell proliferation, differentiation, and apoptosis induction. B cells, TCD4, TCD8-activated cells, neutrophils, monocytes, macrophages, NK cells, and dendritic cells all contain this receptor (18, 19). It modifies the genes of chromatin-modifying enzymes through direct and indirect interactions (20).

The VDR gene, which has 10 introns and 11 exons, is located on chromosome 12. It contains more than 900 SNPs, according to the literature. The DNA binding domain is encoded by exons 2 and 3, where the majority of these polymorphisms accumulate. Exon changes result in alterations to this domain and impact the structure of the receptor, which precludes vitamin D binding (21). Vitamin D receptors molecular signaling is somehow complex. The VDR binds to retinoid X receptors to form a complex, which is what makes up the traditional route. By attaching to the VDR-RXR, vitamin D controls the expression of many genes. VDR could, however, manifest as an intra-membranous receptor. These are the main substances that initiate the alternate routes that activate the cytochromes. Secosteroids are among the chemicals that are produced by the downstream enzymes. The metabolites modulate the function of some transcription factors and alter gene expression (22, 23).

## Vitamin D and the innate immune system

**Figure 1:**
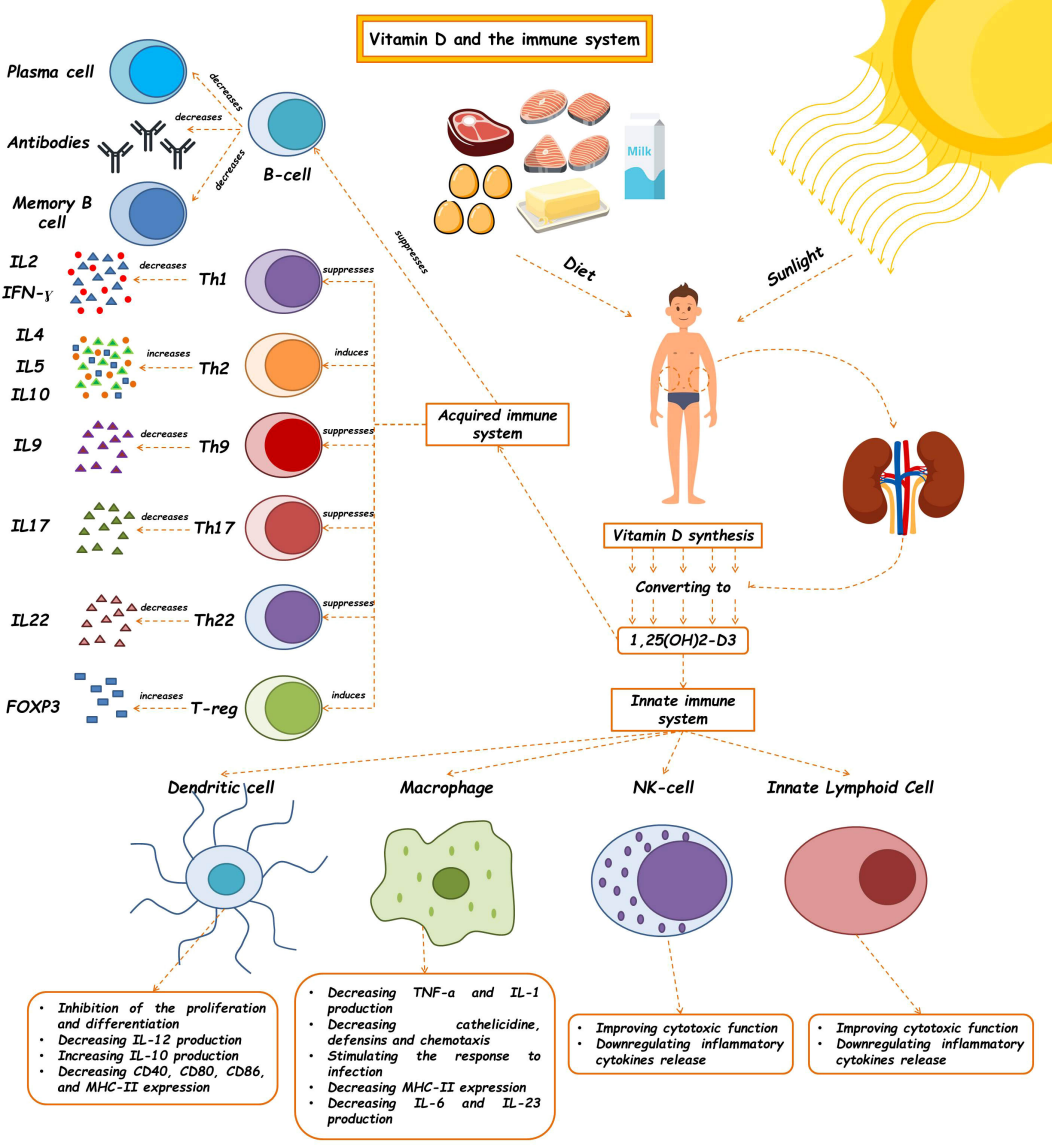
The effects of vitamin D on the immune system. Vitamin D and 1,25(OH)2D3 modulate the innate immune response. The regulatory role of this molecule has been shown to affect the innate immune system, such as macrophages, dendritic cells, Nk cells, and ILCs, so, as a critical molecule plays a role in many diseases' pathophysiology. This vitamin also contributes to making an acquired immune response. (See text for additional details). Th, T helper cell; IL, Interleukin; IFN-γ, Interferon-γ.

The innate immune system, which is the body’s first line of defense against pathogens, is in charge of quick reactions, pathogen detection, and elimination to stop an illness from getting worse. Vitamin D has a crucial role in innate immunity by stimulating the production of pattern recognition receptors (PRRs), antimicrobial peptides, and cytokines in the cells. Additionally, it can prevent the maturation and activation of dendritic cells as well as the differentiation of monocytes into macrophages. Cathelicidins, alpha- and beta-defensins, and other cationic antimicrobial peptides make up the majority of the immune system. The primary function of vitamin D signaling is to control innate immunological responses, according to numerous studies, especially those conducted in recent decades (24). The production of antimicrobial peptides by intestinal epithelial cells, Paneth cells, monocyte/macrophages, and neutrophils is one of the key factors in this control (24). Since the middle of the nineteenth century, vitamin D has been known to induce the antibacterial activity of human monocytes and macrophages (25). The phagocytic and chemo-like activity of macrophages is enhanced by 1,25-(OH)2 D3 (26). The generation of antimicrobial cathelicidin peptides is stimulated by the activation of the toll-like receptor in monocytes and macrophages, which also results in the positive expression of the VDR and alpha 1-hydroxylase genes, killing intrathecal Mycobacterium tuberculosis (27). However, 1,25-(OH)2 D3 suppresses the expression of TLR2 and TLR4 genes in macrophages. After 72 hours, this situation frequently takes control and negatively affects TLR activation and inflammation during the late infection stage. The conversion of 25-OH D3 into 1,25-(OH)2 D3 occurs during infection as a result of increased CYP27B1 expression in activated macrophages and monocytes brought on by cytokines like interferon- γ (IFN- γ) along with toll-like receptor signaling. The 1,25-(OH)2 D3 is then used to enhance the antibacterial activity of macrophages and monocytes via the VDR-RXR pathway. The outcome is an increase in cathelicidin production. The microbial membranes of invasive bacteria and fungus are destabilized by this peptide (28). As a result, 1,25-(OH)2 D3 is immunomodulatory in Mycobacterium TB infection (29). Antimicrobial peptides including 2-defensin and cathelicidin are activated by the calcitriol complex, retinoid X receptor, and VDR (30). Human cathelicidins, such as hCAP18 (either its 17-kDa (140 amino acids) or 5-kDa (37 amino acids) forms) and Leucine-leucine-37 (LL-37), isolated from a large prepropeptide in immune cells (neutrophils) or non-immune tissues (like testis), act against bacteria, fungi, and viruses as a response to infections and destroy microbial membranes (31). Vitamin D signaling has been shown to influence the physiological intestinal tract, aid intestinal hemostasis, and regulate microbiota in healthy individuals (24). Innate lymphoid cells (ILCs) and NK cells are also affected by 1,25-(OH)2 D3. T cell and dendritic cell (DC) responses are modulated by NK cells, which are crucial components of the innate immune system (32). ILCs are a crucial component of immunity. All mucosal tissues, particularly the colon, contain these cells. They are also among the first immune cells to promote cell growth, the healing of wounds, the release of anti-inflammatory mediators in response to infections, and the secretion of antimicrobial peptides (33–35). Through their VDR receptors, 1,25-(OH)2 D3 enhances the cytotoxic activity of NK cells and ILCs. The expression of their inflammatory cytokines is similarly decreased (**Figure 1**) (24, 36–40) (**Table 1**).

**Table 1:** Effects of 1, 25(OH) 2D3 on innate immunity.

Cell	Effects of 1,25(OH)2D3	References
Dendritic cells	↓ proliferation ↓ differentiation ↓ maturation ↓ CD40, CD80, CD86, MHC class-II: decreased T cell stimulation ↓ IL-12; Indirect Th1 response inhibition ↑ IL-10 and Foxp3: Treg induction ↓ Th17 cell induction	(41, 42)
Macrophages	↓ IL-6 and IL-23: decreased Th17 response ↓ TNF and IL-1 ↓ MHC class-II: ↓ antigen presentation ↑ cathelicidins, defensins, phagocytosis, chemotaxis ↑ Stimulation of response to infection ↓ TLRs 9/4/2	(41, 42)
NK-cells	↑ cytotoxic function ↓downregulating inflammatory cytokine expression	(24, 43)
Innate lymphoid cells	↑ cytotoxic function ↓downregulating inflammatory cytokine expression	(24, 44)

Upward arrow means increasing. Downward arrow means decreasing.

## Vitamin D and the acquired immune system

Although the active form of vitamin D leads to the stimulation of innate immune responses, this vitamin causes the suppression of acquired immune responses (**Table 2**). The most significant secretors of IL-2 and interferon-γ (IFN-γ) are T-helper 1 (Th1) cells, induced by the activation of the T cell receptor (TCR) and CD4+ membranous protein. In fact, Th1 is a key player in stimulating other inflammatory leukocytes and serves as a marker of cellular immunity activation. 1,25-(OH)2 D3 inhibits the synthesis of the pro-inflammatory cytokines IL-2 and IFN γ as well as Th1-mediated responses. By inhibiting the nuclear factor of activated T cells (NFAT) and activator protein-1 (AP-1) factors, 1,25-(OH)2 D3 encounters this inhibitory action (49). Additionally, 1,25-(OH)2 D3 increases the production of Th2 anti-inflammatory cytokines (including IL-3, -4, -5, and -10), while decreasing the production of Th9 (IL-9) and Th22 (IL-22) pro-inflammatory cytokines (48, 50). IL-9 is essential for attracting immune cells to the sites of inflammation, particularly mast cells. It is mostly produced by Treg cells. Th9 cell release of IL-9 may be the cause of allergic reactions and inflammatory reactions. The majority of IL-22-producing body cells are still Th22 cells. This crucial interleukin promotes the proliferation and development of keratinocytes. IL-22 enhances the secretion of anti-microbial peptides in the gut epithelium, which may result in a more effective defense against encroaching microorganisms. The anti-inflammatory function of T-regulatory cells, which is crucial for autoimmune control and homeostasis, is induced by 1,25-(OH)2 D3. The primary transcription factor in this class is Forkhead Box P3 (Foxp3). It is essential for 1,25-(OH)2 D3’s transcriptional upregulation as well as for the maturation, maintenance, and function of Tregs (51). It has been suggested that 1,25-(OH)2 D3 in both *in vivo* and *in vitro* conditions increases the amount of Tregs to reduce autoimmunity (52). In general, 1,25-(OH)2 D3 suppresses the induction of T-helper 1 (Th1)-cell cytokines, particularly IFN-γ; however, it also enhances Th2-cell immunological responses, which are mediated both directly by raising IL-4 production and indirectly by suppressing IFN-γ production (53). Many autoimmune and inflammatory disorders, including systemic lupus erythematosus, multiple sclerosis, and rheumatoid arthritis, are caused by Th17 cells, a type of inflammatory lymphocytes that promote inflammation and phagocytosis, notably neutrophils. Through mechanisms such as inhibition of the NFAT and RORt transcription factors, recruitment of histone deacetylase (HDAC), Runt-related transcription factor 1 (Runx1), and a direct impact on induction of Foxp3, the complex of vitamin D/VDR would result in the suppression of IL-17 production (54). Additionally, it may result in T cell homing at inflammatory areas and in the skin via CCR5 and CCR10, respectively. The homeostasis of B cells is directly impacted by VDR expression in their membrane. The active form of vitamin D causes apoptosis in antibody-producing cells, neutralizing the production and differentiation of B cells into plasma cells and memory cells, reducing the production of antibodies, and increasing the homing of these cells through CCR10 to the skin (53). The clinical significance of this influence on B cells can be shown in autoimmune illnesses including those linked to autoreactive antibodies. B cells produced a variety of autoantibodies. These have the power to harm the organs and start an inflammatory reaction that leads to autoimmune diseases. Additionally, a large number of regulatory B cells have been found to suppress the production of autoantibodies (55). In reality, the biologically active form of vitamin D suppresses B cell proliferation and controls their responses. Through lowering stimulant molecules like CD40, CD80, CD86, and MHC class-II in cells presenting antigens like dendritic cells, this vitamin also leads to T cell suppression and immune response modification (**Figure 1**) (41). Let us explore the specific roles that vitamin D plays in some key immune-related disorders in the present review (**Table 3**).

**Table 2:** Effects of 1, 25(OH)2D3 on adaptive immunity.

Cell		Effects of 1,25(OH)2D3	References
B cells		↓ proliferation ↓ Plasma cell development ↓ Antibody secretion ↓Memory B cell differentiation ↑CCR10: Homing to skin	(45)
T cells	Th1	↓ IL‐2 transcription ↓ IFN-γ transcription	(46)
	Th2	↑Th2 Cytokines(IL4,IL5,IL10)	(47)
	Treg	↑Treg cell differentiation ↑ Foxp3 transcription ↑IL10,CTLA4	(46)
	Th17	↓ IL ‐17,IL21 transcription	(46)
	Th22	↓IL22	(48)
	Th9	↓IL -9	(48)

Upward arrow means increasing. Downward arrow means decreasing.

**Table 3:** Summarization of vitamin D function in different immune related disorders.

Disease	Cells affected by vitamin D	Mechanism of action	Target genes	Ref
Allergic rhinitis	T-helper type 2, Eosinophil	Decreased expression of CD86	MIR17HG miR-17-92a-1 cluster host gene	(56, 57)
Asthma	CD4 + T cells	Respiratory infection prevention via enhancing the immunity of the lungs, inhibiting steroid resistance by increasing the production of IL-10	VDR gene	(58, 59)
Atopic Dermatitis (AD)	Keratinocytes	Regulation of epidermal function and local immune response	CYP24A1	(60, 61)
Rheumatoid Arthritis	T cells	Decreasing the production of IL-12 via NF-kB downregulation, inhibition of IFN-γ	TaqI	(62, 63)
Multiple sclerosis (MS)	Whole body	HLA-DRB*15 upregulation	HLA-DRB1	(64)
Parkinson disease (PD)		Alterations in VDR expression levels	BsmI, FokI	(65)
Infection-related disorders	Macrophages, monocytes, Paneth cells	Enhancement of cell autophagy, release of antimicrobial proteins	mTOR, cathelicidine	(66, 67)
Cancer	Tumor cells	MAPK and Nf-kB inhibition, immune regulation	MKP5, Nf-kB	(9, 68)
Diabetes mellitus (DM)	DCs and macrophages, cytotoxic T cells	Self-tolerance and immune regulation	MHC-II and co-stimulatory molecules	(69, 70)
Inflammatory bowel disease (IBD	B cells, T cells, dendritic cells, and macrophages	Enhancing the expression of IL-10, suppressing the proliferation of B and T cells	VDR, IL-10, Cathelicidine	(50, 71)

## Allergic rhinitis

Allergic rhinitis (AR) is a nasal mucosal disease characterized by an allergen reaction due to IgE production (72). The role of vitamin D is explained in multiple investigations related to this disorder. Examining the relationship between vitamin D serum levels and allergic diseases in adults using the third National Health and Nutrition Survey (NHANES III), Wjst et al. noted that AR worsened in vitamin D-deficient patients (73). Another Finnish study tried to investigate maternal intake of vitamin D in the course of pregnancy and its association with asthma and allergic rhinitis incidence in newborns. They recruited 1,669 children affected by allergic rhinitis, atopic eczema, and asthma. It was discovered that high levels of vitamin D intake in pregnant women were related to an allergic risk reduction in 5-year-old children (74). Additionally, Chen et al. suggested that prenatal vitamin D sufficiency has a protective effect on developing allergic rhinitis and aeroallergen sensitization at childhood (75).

Furthermore, pieces of evidence from clinical investigations demonstrated that AR risk can be inversely related to the serum vitamin D level (76, 77). On the contrary, Feng et al. indicated no causal association between them. This study goes against the previous claims of vitamin D supplementation’s protective effects on allergic rhinitis (78). In addition, Searing et al., in an *in vitro* study, explained that this important vitamin may affect dexamethasone’s stimulation on circulatory mononuclear cells’ IL-10 and mitogen-activated protein kinase phosphatase-1 (MAPKP-1). Vitamin D levels reflect a negative correlation with the dose of the inhaler, oral of steroids. The induced mRNA amount of mitogen-activated protein kinase phosphatase-1 (MKP-1) and IL-10-induced vitamin D with dexamethasone was significantly higher than those induced by dexamethasone by itself. MKP-1 and IL-10 are critical for glucocorticoid-dependent anti-inflammatory and repressive effects (79). Also, according to other *in vitro* studies, there is growing evidence that serum vitamin D level is correlated with inflammatory factors. Eosinophil infiltration, allergic symptom scores, and mRNA levels of IL-4 and IL-13 were all lower in the mice group under vitamin D supplementation. Moreover, CD86 expression in the cervical lymph nodes was decreased among T-helper type 2-mediated inflammation and CD11c^+^major histocompatibility complex II-high (MHCII high) cells. IL-4 was also conversely correlated with vitamin D levels (56, 80).

The MiR-17-92 cluster is a human miRNA located on chromosome 13. Increasing the expression of this miRNA has been reported in many immune disorders such as cancers and some allergic diseases, but the regulatory mechanism is not well defined. One way to treat allergic diseases is specific immunotherapy. It has been noted that vitamin D raises specific immunotherapy effects on MiR-17-92 cluster suppression in peripheral B cells in allergic rhinitis patients (81). This study confirmed that vitamin D improves the anti-inflammatory function of corticosteroids in patients with allergic rhinitis. Regarding the effects of vitamin D supplementation in the treatment regimen of allergic rhinitis, Bakhshaee et al. concluded that it could relatively improve symptoms of clinical AR, further emphasizing vitamin D supplementation as a therapeutic agent (82).

## Asthma

The role of vitamin D in asthma is still uncertain. The CDX2 polymorphism is in the 9,913th position in the promoter region of the VDR gene emerging from the substitution of A with G. It has been mentioned that this polymorphism changes the levels of VDR transcription and its overall activity. It also subsequently modulates the genes effective in inflammation, regulation of immunity, and airways remodeling. This polymorphism in its homozygous form has been associated with the diagnosis of asthma and decreased FEV1 (21).

Some cross-sectional studies have indicated a possible relationship between asthma and vitamin D clinically (83). Studies have concluded that a drop in serum level of 1,25-(OH)_2_ D3 was associated with an advance in prevalence, hospitalization, the number of emergency visits, respiratory rate, and a reduction in lung function in children with asthma (84). Recent clinical trials have proved the protective effect of vitamin D supplements in asthmatic patients (85–87). One study demonstrated that weekly oral calcifediol supplementation in vitamin D-deficient adults could help control the disease course and improve patients’ quality of life (88). One study by Gapta et al. reported that the low serum levels of vitamin D in children with steroid-resistant asthma (STRAs) are characterized by decreased lung function, increased corticosteroid use, and asthma exacerbation. The possible reason was that low vitamin D levels help smooth muscles of the respiratory tract to grow and diminish lung function in severe asthma (89). Examples of vitamin D activity in asthma include improving the immune function of the lung tissue and preventing the development of respiratory infections (85, 90, 91) or overcoming resistance to steroids by increasing the production of IL -10 via CD4 + T cells (92). In contrast with the asthma prevention duty of vitamin D, it can cause the exacerbation of it. Low levels of vitamin D (<30 ng/mL) increase the risk of asthma (83, 93).

Furthermore, several studies could not show a significant correlation between vitamin D levels and the incidence of asthma in children and adults (94–96). In addition, several clinical trials failed to find a significant association between vitamin D supplementation in deficient individuals and improvement in clinical outcomes such as controlling the disease course, lessening symptoms and side effects, improving respiratory volumes and ratios, reducing exacerbation episodes, and decreasing required medications (97–99). In addition, vitamin D intake during pregnancy enhances the risk of asthma in children and adults (100, 101).

## Atopic dermatitis

Among the factors involved in atopic dermatitis (AD), the growing importance of vitamin D deficiency was noted in atopic patients. Furthermore, vitamin D is correlated with the antimicrobial peptide (AMP) production by keratinocytes. Vitamin D and its analog play a vital role in treating AD, psoriasis, vitiligo, and acne (17, 102–104). Few investigations have surveyed the prevalence and severity of AD in people with vitamin D deficiency. Atopic dermatitis is associated with vitamin D levels, and vitamin D deficiency increases the risk of atopic dermatitis (105).

On the other hand, it has been noticed that vitamin D levels were higher in mild AD patients than in those with moderate to severe dermatitis (95). It seems that children born to mothers who have a low intake of fish or vitamin D during pregnancy have a higher risk of developing atopic dermatitis symptoms (106, 107). Also, as Wang et al. suggested, low breast milk vitamin D levels may influence the infant’s immune system, inversely correlated with persistent AD symptoms (108).

However, despite the positive relationship between hypovitaminosis D in the above studies and the prevalence or severity of AD, several texts have shown the reverse association (76, 109). Another investigation discovered that children of mothers with elevated serum 25-OH D3 levels had a higher risk of emerging advanced eczema at 9 months and 9 years old (100). Moreover, research carried out by Tian et al. claimed that the risk of atopic dermatitis (AD) in children at the first year of age is generally increased with higher maternal serum vitamin D levels throughout pregnancy (110). From the above considerations, it can be implied that this is a very controversial topic. Vitamin D is sometimes a protective agent and sometimes a risk factor for AD. However, it seems that at least in the majority of studies, it has been suggested that there is a reverse relationship between vitamin D serum levels (food intake or sunlight) and AD prevalence and severity. Further fueling the conflicting evidence in this matter, Lucas et al. believed that 1-hydroxylase CYP27B1 (responsible for 1,25-Vitamin D3 synthesis), the vitamin D receptor, and the vitamin D-mediated signaling target gene CYP24A1 are all upregulated as a result of allergic sensitization, which subsequently auto-downregulates vitamin D receptor-mediated signaling through reducing available ligand accumulations. This study suggested that an increase in local vitamin D-mediated signaling is a pro-allergic condition at the inflammation site, which results in low serum levels of vitamin D (111).

Vitamin D plays an essential role in regulating innate and acquired immune mechanisms. Vitamin D receptors (VDRs) have been found in various cells, including keratinocytes and multiple immune cells (112). 1,25-(OH)_2_ D3 prevents T cell proliferation, especially Th1s producing IL-2 and interferon-γ leading to macrophages and Th17s activation. It induces production of IL-17 and IL-22 (113–115). Moreover, 1,25-(OH)_2_ D3 reproduce CD4 +/CD25 + cells through stimulating IL-10 production, which ultimately impairs the Th1s and Th17s development (116, 117).

The polymorphisms of the VDR gene may cause differences in response to vitamin D in inflammatory conditions. Some of them might be discovered in severe AD patients, such as rs2228570, rs1544410, rs7975232, and rs731236. This indicates that the VDR controls AD by regulating epidermal function or affecting the local immune response (60). Langerhans and inflammatory dendritic epidermal cells (IDEC), both located in the epidermis of patients suffering from AD, expressing FcϵRI (high-affinity IgE receptor) and sensing allergens. Herrmann et al. explained that active vitamin D3 could downregulate the receptor at the protein and mRNA levels of its α-chain, impairing IgE-mediated inflammatory processes afterward (118). Cristi et al. also concluded that vitamin D sufficiency lowers the allergic phenotype of circulating DCs in AD children (119). As Mansour et al. suggested, vitamin D supplementation can be regarded as an effective treatment, decreasing the risk of severe atopic dermatitis symptoms, adding it to the existing studies already confirming vitamin D adjuvant therapy to be beneficial in mild to moderate cases (120).

## Rheumatoid arthritis

Rheumatoid arthritis (RA) is an autoimmune disease of connective tissue that mainly affects synovial joints, causing grueling pain and reducing life expectancy (121). Although the exact cause is not entirely understood, researchers have suggested that genetic and environmental factors contribute to the RA pathogenesis (122). Considering the suppressant effects of vitamin D and the possible association between vitamin D deficiency and autoimmune disorders (123), it has been studied as a potential aid to the pathogenesis of autoimmune diseases, including RA (124). Studies have shown that many autoimmune disorders are correlated with lacking vitamin D (125, 126). This is confirmed that 1,25-(OH)_2_ D3 reduces the production of IL-12 production through NF-kB downregulation, inhibits the secretion of IFN-γ, and limits the expression of IL-6 receptor; *in vivo* administration of 1,25-(OH)_2_ D3 appears to have preventive effects on autoimmune diseases (77, 127, 128). Also, by inhibiting the expression of macrophage aromatases, vitamin D can reduce the conversion of androgens to estrogens which play a significant part in the activation of B lymphocytes and consequently the autoimmune response in RA (62).

The activity of vitamin D depends on VDR, and the activation of VDR can inhibit pro-inflammatory T cells and DC differentiation. In addition, VDR agonists induce T-regulator and NK cells and thus suppress self-immunity (63). It has been proved that VDR polymorphism accelerates RA sensitivity (129). One of these polymorphisms is TaqI or rs731236, located within Exon 9 at the 3′ end of the VDR gene. Probably the TT genotype of this polymorphism is a risk factor for RA (130). Studies have indicated that high levels of vitamin D absorption can reduce the risk of rheumatoid arthritis by 24% (131). In addition, vitamin D deficiency in these patients can lead to a more severe and active course of the disease; thus, vitamin D serum level may be a predictive factor for one-year disability and disease progression (132, 133). But not all evidence is in favor of the preventative effects of vitamin D. A recent study on RA patients concluded no significant differences between vitamin D-deficient and -non-deficient patients considering criteria such as swollen joints count (SJC), VAS-pain, tender joints count (TJC), and DAS28 scores (134). Further investigations can shed light on various features of the relationship between RA and vitamin D.

## Multiple sclerosis

The pattern of MS occurrence varies throughout the world: its prevalence shows a steep slope over the equator, and the disease is more prevalent in areas near the poles (135). This can be associated with the level of UVB exposure (136) and can support the theory that environmental factors frequently influence the MS risk in early life. Studies have determined a relationship between the serum level of 25-OH D3 and MS risk development, namely, and there is a negative association between vitamin D levels and MS (137, 138). Obesity and smoking remain the two other MS risk factors correlated with vitamin D deficiency (139, 140). Although smoking and obesity may affect the MS risk by changing the vitamin D level, they may also affect the risk of MS in a vitamin D-independent way (141, 142). The association between MS and five loss-of-function mutations on the CYP27B1 gene encoding the 25-OH vit-D-1a-hydroxylase enzyme strongly indicates vitamin D’s critical role in MS etiology has been confirmed that these mutations decrease the level of vitamin D (143). However, three subsequent attempts to repeat this finding were unsuccessful. Now it seems that although these variants are associated with the risk of developing MS, their contribution to MS inheritance is small (144–146). Furthermore, the expression of HLA-DRB1*1501, the most potent genetic marker for MS, is regulated through the VDRE (vitamin D response element) in the promoter region (147, 148). Further investigations have revealed that vitamin D receptor gene polymorphisms could affect vitamin D absorption and increase MS susceptibility in various populations (148, 149). According to the meta-analysis conducted by Imani et al., Taql and Bsml polymorphisms, despite Apal polymorphism, lead to MS risk enhancement (150). Another risk reduction theory was explained in the study by Spanier et al. It stated that CTLA-4 functions as a mediator for MS prevention and, in the presence of vitamin D, its expression is significantly increased inside intracerebral myeloid cells (151).

More than 200 SNPs were detected out of the HLA region during the GWAS studies which are significantly related to MS disease. Among them, some SNPs are associated with genes responsible for vitamin D metabolisms such as rs2248359, CYP24A1, rs2248137, rs12368653, rs703842, rs10876994, CYP27B1, rs201202118, rs703842, and rs701006 (152, 153). These observations indicate that vitamin D relates to the transcription of the genes associated with MS and alters their mechanism of action (154). This important nutrient is assumed to have a key role in repairing genes expression, especially MYH, OGG1, MTH1, and NRF2 in MS patients (155).

## Parkinson disease

Parkinson’s disease (PD) is a motor disorder characterized by tremor, stiffness, acne, and loss of local reflexes, leading to immobility and frequent corrosion. There were pieces of evidence that manifested vitamin D deficiency can increase the prevalence of PD (156–158). Interestingly, people with high concentrations of vitamin D in their serum show a reduction in Parkinson’s risk (159). This is also related to exposure to ultraviolet radiation (160). Many studies have proved that vitamin D is critical for the growth and function of the brain. VDRs and 1-alpha-hydroxylase, the enzyme responsible for vitamin D activation, are observed in the substantia nigra and the hypothalamus’s principal neurons and glial cells (161). Mice whose VDRs have been knocked out have developed muscular and motorized dysfunctions (162). In addition, higher levels of 1,25-(OH)_2_ D3 and VDR FokI CC genotypes are associated with mild forms of Parkinson’s disease (163). Different VDR polymorphisms such as the DHCR7/NADSYN1 locus and the CYP2R1 gene can affect clinical symptoms of PD through an impaired mechanism of turning vitamin D into valuable chemicals (164).

Recent studies have demonstrated the association between BsmI and FokI polymorphisms in VDR and PD sensitivity (165, 166). The BsmI bb genotype is more common in PD and causes the alteration in the VDR expression levels of mRNA. In patients with PD, FokI CC and TC genotypes are more prevalent than TT. In those regions with higher UV radiation, an association between UV and TaqI and ApaI has been found. The PD risk is reduced in the homozygous form of the TT TaqI genotype and the homozygous form of the GG ApaI genotype (167).

## Infection-related disorders

Vitamin D plays an important role in regulatory actions of the immune system. This is essential for controlling infection-related disorders. Prior investigations indicated that respiratory infections, human immunodeficiency virus (HIV), and Mycobacterium tuberculosis (TB) were strongly associated with low levels of vitamin D in the circulation (168). The major antimicrobial protein, cathelicidin, is known to be necessary in the defensive actions of monocytes against the invading pathogens (66). Vitamin D receptor signaling crosstalks with the production of cathelicidin in these cells (169). Also, vitamin D-cathelicidin axis is vital in intestinal inflammation preventing gastrointestinal infections (170). The enhancement of the Paneth cells in response to the vitamin D receptor signaling was also studied very much (171). Toll-like receptors 1/2 (TLR 1/2) have been identified to be activated by 1,25-dihydroxy-vitamin D (vitamin D3) inducing the antimicrobial effects of tissue macrophages (27). By means of mammalian target of rapamycin (mToR) signaling, this micronutrient is also capable of inflammation attenuation in airway systems through enhancing cell autophagy (67).

Vitamin D supplementation and prevention of the infections such as coronavirus disease (COVID-19) has recently been investigated by many scientists (172–174). The results are still conflicting. Regarding TB infection, a large randomized clinical trial was conducted with a sample size of 8,851 children. The conclusion stated that vitamin D had no superior effect on TB infection risk than placebo (173). On the other hand, some studies demonstrated that vitamin D supplementation had a promising lowering impact on the risk of upper respiratory infections (172, 174). There remains a huge demand for larger and more accurate studies in order to assess the net effect of vitamin D on the incidence of various infection types.

## Cancer

Cancers are among the most common and important immune system-related disorders in the world. They emerge from the dysfunction occurring in cell proliferation, differentiation, and apoptosis (9, 175). The association between the serum level of vitamin D and the risk of various cancers has been investigated several times in the past. Colorectal cancer (176), breast cancer (177), skin cancer (178), prostate cancer (179), liver cancer (180), and head and neck cancers (181) are some of the examples. According to Ma et al.’s study, high blood levels of vitamin D was correlated with a 33% reduction in the risk of colorectal cancer (182). Regarding head and neck neoplasms, high vitamin D serum levels decreased the incidence by 32% and also lowered the mortality risk in these patients (183).

VDR is upregulated in many tumor cells of different types of cancers. For instance, epigenetic studies indicated that in colorectal cancers, the adipose tissue overexpressed VDR. As we probably know, adipose tissue dysfunction is a vital mechanism underlying the emergence of colorectal cancer (184). This overexpression has been observed in prostate and ovarian cancers, too (179, 185). However, prior investigations have suggested that in some cases more VDR expression in tumor cells might lead to a better prognosis and treatment response (186, 187). Inflammation and immune response remained a matter of discussion in cancer progression and spreading (188). As mentioned, vitamin D has been identified to have a major impact on the regulation of inflammation, especially in microenvironments. The signaling might be activated or inhibited through two main pathways: MAP Kinase Phosphatase 5 (MKP5) and Nf-kB (9). The existing evidence revealed that vitamin D might upregulate the MKP5 in tumor cells causing MAPK inhibition and, following that, decreased production of IL-6 (189, 190). This resulted in the regulation of inflammatory response of the immune cells. Additionally, previous articles have stated that this important micronutrient could inhibit the activity of Nf-kB as the upstream compounds of another pro-inflammatory cytokine, IL-8, production (191). Also, the binding of Nf-kB to the DNA might be disturbed in the presence of vitamin D (68).

Concerning the use of vitamin D supplementation in preventing or treating distinct types of cancers, many randomized or non-randomized clinical trials have been conducted (9). A large randomized clinical trial with 25,871 patients revealed that vitamin D supplementation did not influence the incidence of invasive cancers (192). Also, another study on healthy postmenopausal women confirmed the inability of vitamin D in cancer prevention (193). On the other hand, some studies have suggested that vitamin D might improve the survival rate in patients suffering from cancer. Akiba et al. achieved favorable results regarding non-small cell lung cancers (194). Further large multicenter trials are absolutely required in order to finally build a consensus about this major issue.

## Diabetes mellitus

Type 1 diabetes (T1D) is a prevalent disease across the globe with a combination of factors (genetic and environmental) as its causes. It is considered an autoimmune disease induced by a T cell-mediated mechanism in which dendritic cells (DC) and different pro-inflammatory cytokines play vital roles (195–197). On the other hand, studies are getting increasingly confident in the role of vitamin D deficiency in different autoimmune diseases (198–200). Therefore, vitamin D is discussed in many studies as a protective factor in developing T1D (201, 202).

The seasonal pattern of T1D and its various prevalence depending on latitude degree and ultraviolet B (UVB) irradiance support this association theory (203–209). Epidemiological studies show a trend of higher T1D incidents in adults and children suffering from vitamin D deficiency (196, 210–212). In addition, it is stated that there is an association between several polymorphisms of VD-related genes and T1D (196). Polymorphic expression of genes encoding vitamin D metabolizing enzymes, CYP27B1 as well as CYP2R1 and 7-dehydrocholesterol reductase (DHCR7), unlike CYP24A1 gene expression, has been suggested to be associated with T1D susceptibility (213–215). Although there is conflicting evidence regarding the association of VDR polymorphism with T1D (216, 217), Zhang et al. indicated that at least one of four VDR gene polymorphisms is linked with a higher possibility for T1D (218).

Multiple factors are involved in the vitamin D level impacting the immunopathogenesis of T1D and affecting both innate and acquired immunity (17, 195). During T1D development, islet autoantigens appear at the antigen-presenting cells (APCs) surface, including DCs and macrophages, which induce a cytotoxic T cell response (196). Vitamin D is produced by dendritic cells (DC) and also applies immunomodulatory effects toward self-tolerance in these cells (69, 219). As a matter of fact, almost all of the immune system cells express the VDR (220). The active form of vitamin D prevents the maturation of DCs. As a result, surface expression of major histocompatibility complex (MHC)-II and co-stimulatory molecules is hampered, and antigen presentation and T cell activation are stopped (70). Furthermore, this active form induces mature DCs apoptosis. Also, it differentiates them into a tolerogenic state. Therefore, regulatory T cells (Treg) are induced to a greater extent (221–223). Moreover, Mauf et al. suggested that 25-OH D3 shows its immunomodulatory effects by differentiating monocytes less into DCs and more into intermediate cells with similar phenotypes to those of tolerogenic DCs (69). Besides this indirect effect of vitamin D on T cells, they can be targeted directly, too (196). It is worth mentioning that vitamin D can also directly affect pancreatic beta cells and their insulin secretion, therefore making them more resistant (196).

As mentioned, vitamin D deficiency is considered an environmental risk factor for developing autoimmune diseases like T1D (212, 224). Therefore, many studies have evaluated its potential preventive or therapeutic effect (199, 210). It is demonstrated in animal models that the active form of vitamin D can perform immune modulation in diabetes-prone mice in several ways. Increasing Tregs, triggering a Th1/Th2 shift in the islets and the pancreatic draining lymph nodes, preserving defective T cell selection in the thymus, and eliminating apoptosis-resistant T cells are all parts of that immunomodulatory action (225–229). Apart from this non-antigen-specific immunomodulation, antigen-based immunotherapies for T1D are also reported (224). Several studies have discussed the protective immunological effect of vitamin D3 in T1D patients, especially T cell modulation (212, 230). Gabbay et al. reported a significant increase in Tregs upon 12 months of vitamin D3 supplementation in patients with recent-onset T1D (231). Also, Treiber et al. observed a significant increase in the suppressive function of Tregs after vitamin D3 supplementation for 12 months (232). Contradictory to these results, some studies show no meaningful protective effect of vitamin D (202, 233–236). That makes it crucial for future studies to better evaluate the disease intervention by assessing the administering of vitamin D, its exact compound choice, the dosage needed, and the ideal treatment regimen (224).

## Inflammatory bowel disease

Inflammatory bowel diseases (IBD) are a group of chronic immune-mediated diseases, with ulcerative colitis and Crohn’s disease being the most renowned ones. Several studies have shown that the prevalence of vitamin D deficiency in patients with IBD is higher than in a healthy population (237–242). Although it has not been precisely determined that this difference is a cause or consequence of the disease, it has drawn attention to the possible impacts of vitamin D on the etiology and course of the disease. In addition to human studies, *in vitro* and *in vivo* model analysis put forward three possible mechanisms that vitamin D may affect the onset and progression of IBD.

Gut microbiota may be another target for vitamin D to ameliorate the IBD course or even prevent its onset (243, 244). These bacteria exert their effects through different metabolites. Butyrate is one of these metabolites, which, in addition to being an energy substrate for mucosal cells, accelerates epithelium healing (245, 246). Lithocholic acid, another bacterial metabolite, suppresses IL-2 production and subsequently decreases inflammation (247). It is worth noting that vitamin D and microbiota have a bidirectional interaction which means microbiota can also utilize vitamin D downstream pathways (248–250).

Vitamin D can target B cells, T cells, dendritic cells, and macrophages throughout innate and acquired immune systems, considering they all express VDR (251). *In vivo* and *in vitro* studies suggest that vitamin D is able to enhance the expression of IL-10 in dendritic cells and the production of cathelicidin by macrophages (71, 252). In contrast, toll-like receptors and IL-12 expression in macrophages and dendritic cells are inhibited by the activated form of vitamin D (29, 253). This inhibition leads to less dendritic cell-induced activation of T cells. Besides, vitamin D can directly suppress B and T cell proliferation (50). Vitamin D was able to interfere in inflammatory/anti-inflammatory production balance, resulting in reduced production of T cell-inflammatory cytokines, including IL-2, interferon (IFN)-γ, IL-17, and TNF-α, and enhanced anti-inflammatory cytokines production such as IL-10 by regulatory T cells and IL-4 by Th2 cells (50, 254–256). Overall, the induction of regulatory cells like Tregs and CD8αα was increased (255, 257).

Various human studies have suggested a positive effect on different outcomes from vitamin D supplementation in IBD patients. Reduced disease activity, decreased risk of postoperative endoscopic recurrence, lower clinical activity score, lower relapse rate, and improved odds of remission were reported by different studies (258–263). One study even reported vitamin D effects on all-cause mortality (264). However, not all evidence is in favor of the preventative effects of vitamin D (265). One RCT concluded no difference in CRP, fecal calprotectin, Crohn’s disease activity index (CDAI), and quality of life between supplemented and control groups (266). At the same time, another RCT showed only a non-significant lower relapse rate in the experimental group (267). Three meta-analyses of different RCTs were conducted. Two established that vitamin D supplementation improves clinical and biochemical disease activity scores, while the third did not report such improvements (261, 268, 269). Further investigations can reveal more aspects of the complex relationship between IBD and vitamin D.

## Conclusion

Studies have shown a strong correlation between vitamin D and the innate and adaptive immune systems, suggesting that low vitamin D levels may contribute to immune response dysregulation. However, in recent years, the precise function of the alternate pathways of vitamin D and its receptor has not yet been sufficiently elucidated. We all need to be aware of what these signaling pathways do in various immune system components. Reviews of the literature revealed that, during the past 40 years, a number of studies have been conducted to suggest the protective effect of vitamin D in autoimmune illnesses. Randomized controlled clinical trials are still lacking, though, in this area. Larger clinical investigations will now be necessary to determine the precise impact of vitamin D supplementation on the pathophysiology of different diseases. Of the immunomodulatory treatments now on the market, they might be as effective as the others. Additionally, the right supplement dosage and manner of administration must be determined. But for present approaches to preventing diseases brought on by compromised immune-homeostasis, vitamin D has emerged as a potential and relatively safe supplement.

## Author contributions

AR: Study design, searching databases, writing manuscript, final revise. AG: searching databases, writing manuscript, final revise. AS: writing manuscript, final revise. BS: writing manuscript, final revise. MM: writing manuscript, final revise. AD: writing manuscript, final revise. GS: study design, supervising all steps, resolving conflicts, final revise. All authors contributed to the article and approved the submitted version.
